# EEMD and Multiscale PCA-Based Signal Denoising Method and Its Application to Seismic P-Phase Arrival Picking

**DOI:** 10.3390/s21165271

**Published:** 2021-08-04

**Authors:** Kang Peng, Hongyang Guo, Xueyi Shang

**Affiliations:** 1 State Key Laboratory of Coal Mine Disaster Dynamics and Control, School of Resources and Safety Engineering, Chongqing University, Chongqing 400044, China; pengkang@cqu.edu.cn (K.P.); guohongyang@cqu.edu.cn (H.G.); 2 School of Resources and Safety Engineering, Central South University, Changsha 410083, China; 3 State Key Laboratory of Coal Resources in Western China, Xi’an University of Science and Technology, Xi’an 710054, China

**Keywords:** signal denoising, principal component analysis, ensemble empirical mode decomposition, microseismic signal, P-phase arrival picking

## Abstract

Signal denoising is one of the most important issues in signal processing, and various techniques have been proposed to address this issue. A combined method involving wavelet decomposition and multiscale principal component analysis (MSPCA) has been proposed and exhibits a strong signal denoising performance. This technique takes advantage of several signals that have similar noises to conduct denoising; however, noises are usually quite different between signals, and wavelet decomposition has limited adaptive decomposition abilities for complex signals. To address this issue, we propose a signal denoising method based on ensemble empirical mode decomposition (EEMD) and MSPCA. The proposed method can conduct MSPCA-based denoising for a single signal compared with the former MSPCA-based denoising methods. The main steps of the proposed denoising method are as follows: First, EEMD is used for adaptive decomposition of a signal, and the variance contribution rate is selected to remove components with high-frequency noises. Subsequently, the Hankel matrix is constructed on each component to obtain a higher order matrix, and the main score and load vectors of the PCA are adopted to denoise the Hankel matrix. Next, the PCA-denoised component is denoised using soft thresholding. Finally, the stacking of PCA- and soft thresholding-denoised components is treated as the final denoised signal. Synthetic tests demonstrate that the EEMD-MSPCA-based method can provide good signal denoising results and is superior to the low-pass filter, wavelet reconstruction, EEMD reconstruction, Hankel–SVD, EEMD-Hankel–SVD, and wavelet-MSPCA-based denoising methods. Moreover, the proposed method in combination with the AIC picking method shows good prospects for processing microseismic waves.

## 1. Introduction

Signals from various practical applications are usually corrupted by unwanted noise, such as background noise, electronic noise, and instrumental noise. Consequently, to improve signal detection, classification, and phase arrival picking, it is necessary to remove unwanted noise. Currently, signal denoising is widely used in the fields of medical signals [1], speech signals [2], mechanical diagnosis [3], structural health monitoring [4,5,6], and seismic monitoring networks [7,8,9]; however, this study focused specifically on microseismic (MS) signal denoising.

Spectral filtering is frequently applied to denoise a signal, and a Butterworth-based filter (low-pass filter, high-pass filter, and band-pass filter) is usually adopted [10], which is simple but may filter out a frequency band that contains useful information when the noise and signal share the same frequency band. In addition, the adopted frequency band may vary for different signals; in other words, it cannot denoise a signal adaptively. Therefore, more effective filtering methods have been proposed for signal denoising, especially based on multiscale features (e.g., wavelet analysis and empirical mode decomposition).

For wavelet analysis-based signal denoising, Beenamol et al. [7] used Shannon and Tsallis entropies to select important wavelet coefficients to denoise a signal; however, noise may still exist in the selected wavelet coefficients. Thus, Tang et al. [8], Mousavi et al. [9], and Chen et al. [11] denoised MS signals based on the combination of wavelet transformation and adaptive thresholding, synchro squeezing wavelet transformation and custom thresholding of single-channel data, and empirical wavelet transformation and automatic noise attenuation thresholding algorithms. However, redundant information in the low-frequency band is not removed by filtering out the high-frequency components and thresholding. Patil [12] combined wavelet transformation and singular value decomposition (SVD) for noise reduction, whereas Bakshi [13] decomposed signals by wavelet transformation and applied principal component analysis (PCA) to denoise the wavelet coefficients of multiple signals containing similar noises and obtained good denoising results. SVD and PCA can efficiently separate useful signals from noise. Wavelet analysis decomposes signals from high- to low-frequency components through wavelet function expansion and translation, which has limited adaptive decomposition abilities for complex signals.

Empirical mode decomposition (EMD), proposed by Huang et al. [14], has better adaptability than wavelet decomposition, and EMD and its improvement have been widely used in signal processing. Mao et al. [15], Dao et al. [16], and Li et al. [17] proposed signal denoising methods based on EMD and thresholding; however, some redundant information could not be denoised by thresholding. Qiao et al. [18] adopted EMD with multiscale eigenvalues of SVD for denoising, which could further reduce noise. However, similar components may exist in different scales for EMD, making it difficult to separate useful components and noise. To overcome this problem, Wu and Huang [19] proposed ensemble empirical mode decomposition (EEMD). On this basis, Jia et al. [20], Han et al. [21], and Li et al. [17] took advantage of permutation entropy, adaptive thresholding, and soft thresholding to denoise signals, attaining a better denoising performance than that based on EMD. Moreover, variational mode decomposition (VMD) has been applied for signal denoising [22], which successfully avoids mode mixing and is robust for both noise and sampling, unlike EMD.

Subsequently, SVD was combined with the Hankel matrix to denoise a signal [23], where the Hankel matrix can expand a signal into a high-dimensional matrix and is very beneficial for feature extraction. Recently, coda wave interferometry (CWI) has been successfully applied to monitor stress distribution and damage detection in engineering [4,5,6], where wavelet transformation [5], normal band-pass filter, narrow band-pass filter, and double-notch filter [6] were used to extract useful signal features. These techniques provided a good research direction for noise reduction and damage detection. In addition, deep neural networks have been proposed for signal denoising; for example, DeepDenoiser [24] achieves impressive denoising of seismic signals, even when the signal and noise share a common frequency band. However, perfect separation of signal and noise in the time–frequency domain requires recovering two complex spectra for the signal and noise from the complex spectra of the noisy signal.

In this study, we focused on the PCA denoising concept proposed by Bakshi [13], which can enhance useful signal and noise separation, and the highly adaptive EEMD, Hankel matrix, and multiscale PCA (MSPCA) are combined to remove noise from a single signal. The main innovations and contributions of this research are as follows: (1) The EEMD was introduced into MSPCA denoising to achieve improved signal decomposition adaptability compared to wavelet decomposition. (2) The Hankel matrix was used to transform a single EEMD component into high-dimensional data that are suitable for conducting PCA denoising on only one signal. (3) Soft thresholding was selected for further denoising of the PCA-denoised component. (4) The denoising results are superior to those of the low-pass filter, wavelet reconstruction, EEMD reconstruction, Hankel–SVD, EEMD-Hankel–SVD, and wavelet-MSPCA-based denoising methods. The remainder of this paper is organised as follows. Section 2 introduces the EEMD, Hankel matrix, and MSPCA-based denoising methods. The synthetic and application tests are described in Section 3 and Section 4, respectively. Section 5 compares the proposed method with other denoising methods. The research results and prospects are presented in Section 6.

## 2. Theoretical Fundamentals

### 2.1. Basis of EEMD

Wu and Huang [19] proposed a noise-assisted EEMD based on EMD by adding white noise to the signals. The steps of the EEMD algorithm are as follows.

(1) By adding a group of white Gaussian noise *ω_j_*(*t*) into a noisy signal *x*(*t*), a new signal $x_{j}^{\prime }\left(t\right)$ is obtained as follows:

(1) $$x_{j}^{\prime }\left(t\right)=x\left(t\right)+\omega_{j}\left(t\right)$$

where *t* is the signal time, *j* is the *j*th time of adding white Gaussian noise, and *j* = 1, 2,…, *M*, where *M* is the number of times white Gaussian noise is added.

(2) Through EMD of $x_{{}_{j}}^{\prime }\left(t\right)$, *n* intrinsic mode functions (IMFs), $c_{j,i}$, and one residual component $c_{j,n+1}$ are obtained as follows:

(2) $$x_{{}_{j}}^{\prime }(t)=\sum_{i=1}^{n}c_{j,i}(t)+c_{j,n+1}$$

where *i* is the IMF identity, *i* = 1, 2,…, *n*, and *n* is the number of IMFs.

(3) By adding different white Gaussian noises [*ω_j_*(*t*)] into the noisy signal *x*(*t*) and repeating steps (1) and (2), the *i*th IMF component $c_{j,i}(t)$ and the residual component $c_{j,n+1}$ through the *j*th decomposition are obtained.

(4) Based on the zero-mean principle of the spectra of white Gaussian noise, the effects of white Gaussian noise as the reference structure of the time-domain distribution are eliminated. The average IMF component *c_i_*(*t*) and the residual component $c_{j,n+1}$ corresponding to noisy signals can be expressed as follows:

(3) $$\left\{ \begin{array}{l} c_{i}\left(t\right)=\frac{1}{M}\sum_{j=1}^{M}c_{j,i}(t)\\ c_{n+1}\left(t\right)=\frac{1}{M}\sum_{j=1}^{M}c_{j,n+1}(t) \end{array}\right.$$

The relationship between the amplitude, times, and denoising effect of the added white Gaussian noise into the noisy signal *x*(*t*) is represented as follows:

(4) $$\varepsilon_{i}=\frac{\varepsilon }{\sqrt{M}}$$

where $\varepsilon_{i}$ is the denoising effect of the added white Gaussian noise, and *ε* is the amplitude of the white Gaussian noise.

As shown in Formula (4), when the noise amplitude is fixed, the larger the number of times white Gaussian noise is added, the closer the final decomposition result is to the true values. If the amplitude of the added noise is too small, noise does not affect the selection of the extreme much, thus playing no small role in complementing the scales.

(5) Finally, the noisy signal *x*(*t*) can be decomposed as follows:

(5) $$x\left(t\right)=\sum_{i=1}^{n}c_{i}(t)+c_{n+1}(t)$$

### 2.2. Basis of Hankel Matrix and MSPCA

For a single signal, only one non-zero singular value corresponds to an SVD. Therefore, a singular value cannot be used for denoising. Hence, we constructed a *p* × *q* order Hankel matrix ***H****_i_* from *c_i_*:

(6) $$H_{i}={\left(c_{i,km}\right)}_{p\times q}=\left[\begin{array}{cccc} c_{i,}{}_{11} & c_{i,12} & \cdots & c_{i,1q}\\ c_{i,21} & c_{i,22} & \cdots & c_{i,2q}\\ \vdots & \vdots & \vdots & \vdots \\ c_{i,p1} & c_{i,p2} & \cdots & c_{i,pq} \end{array}\right]$$

where *c_i_*, *_km_* = *c_i_* (*k* + *m* − 1), and *k* + *m* − 1 = *N*, *k* and *m* are the row and column identities, respectively, and *N* is the total sampling number of *c_i_*.

Based on the SVD, matrix ***H****_i_* can be decomposed as follows: $H_{i}=U\sum V^{T}$, ($U\in R^{p\times p}$, $\sum \in R^{p\times q}$ and $V\in R^{q\times q}$). We marked T=U∑ and P=V. ***H*** can be then treated as the sum of the outer products of the *q* vectors:

(7) $$H_{i}={\mathrm{TP}}^{T}=\sum_{k=1}^{q}t_{k}p_{k}{}^{T}=t_{1}p_{1}{}^{T}+t_{2}p_{2}{}^{T}+\cdots +t_{q}p_{q}{}^{T}$$

where ***t****_k_* and ***p****_k_* indicate the score vector and load vector, respectively, *k* = 1, 2,…, *q*, and T represents the matrix transposition.

The ***t****_k_* length reflects the $H_{i}$ coverage in direction ***p****_k_*: a longer ***t****_k_* represents greater importance, while a shorter ***t****_k_* is mainly noise and placed in ***E***. Subsequently, matrix ***H*** can be rewritten as

(8) $$H_{i}=t_{1}^{\prime }p_{1}{}^{T}+t_{2}^{\prime }p_{2}{}^{T}+\ldots +t_{q^{\prime }}^{\prime }p_{q^{\prime }}{}^{T}+E$$

where $t_{k}^{\prime }$(*k* = 1, 2, ……, ***q’***) is the score vector; a larger ***t****_k_* remains unaltered, whereas a shorter ***t****_k_* is set as a zero vector.

Considering that the PCA of the matrix, $H_{i}$ is equivalent to the eigenvector analysis of the covariance matrix $H_{i}{}^{T}H_{i}$, and the load vector of matrix $H_{i}$ is the same as the eigenvector of $H_{i}{}^{T}H_{i}$. Therefore, we determine $t_{j}^{\prime }$ depending on the eigenvalues of the matrix $H_{i}{}^{T}H_{i}$, and the descending ordered eigenvalues are

(9) $${\hat{\lambda }}_{1}\geq {\hat{\lambda }}_{2}\geq \cdots \geq {\hat{\lambda }}_{q}$$

In general, 85% of the cumulative ratio between an eigenvalue and the total sum of the eigenvalues is selected as the threshold to determine $t_{k}^{\prime }$. The eigenvalues corresponding to the abandoned eigenvectors are set as zero vectors, thus providing the reconstructed Hankel matrix as

(10) $${\hat{H}}_{i}=\hat{T}P^{T}=t_{1}^{\prime }p_{1}{}^{T}+t_{2}^{\prime }p_{2}{}^{T}+\ldots +t_{q^{\prime }}^{\prime }p_{q^{\prime }}{}^{T}$$

The PCA-denoised component $c_{i}^{\prime }$ is obtained from the data of the first row and last column in ${\hat{H}}_{i}$.

### 2.3. EEMD-MSPCA-Based Denoising Method

**Figure 1:**
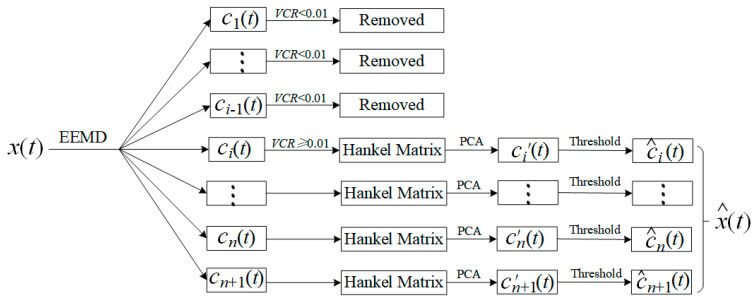
Principle of EEMD-MSPCA-based MS signal denoising.

The first few components obtained by EEMD are mainly high-frequency noises, and noise may exist in the low-frequency components. Therefore, we proposed an EEMD-MSPCA-based seismic signal denoising method (Figure 1), and the specific steps are as follows.

(1) The noisy signal *x*(*t*) is decomposed by EEMD described in Section 2.1, obtaining *n* IMFs $c_{i}(t)$ and one residual component $c_{n+1}(t)$.

(2) The variance contribution rates (VCR) of the IMFs are calculated, and the first few high-frequency components with VCRs < 0.01 are removed. The VCR of the *i*th IMF $c_{i}(t)$ is defined as

(11) $$VCR_{i}=\left[\frac{1}{N}\sum_{t=1}^{N}c_{i}{\left(t\right)}^{2}-{\left(\frac{1}{N}\sum_{t=1}^{N}c_{i}(t)\right)}^{2}\right]/\sum_{i=1}^{n+1}\left[\frac{1}{N}\sum_{t=1}^{N}c_{i}{\left(t\right)}^{2}-{\left(\frac{1}{N}\sum_{t=1}^{N}c_{i}(t)\right)}^{2}\right]$$

(3) The Hankel matrix $H_{i}$ is constructed for each remaining IMF $c_{i}(t)$, which is treated as the data matrix for PCA denoising, and its score vector and load vector are calculated through SVD.

(4) Eigenvalues and eigenvectors of the covariance matrix $H_{i}{}^{T}H_{i}$ are calculated, and the eigenvalues are ranked in descending order. The score vector that corresponds to an eigenvalue with a cumulative eigenvalue percentage larger than 85% is set as a zero vector, and a detailed illustration is shown in Section 3. Subsequently, the Hankel matrix is reconstructed with the new score vector and load vector to obtain the denoised signal $c_{i}^{\prime }(t)$.

(5) The signal $c_{i}^{\prime }(t)$ is further denoised with soft thresholding and calculated as follows:

(12) $$Th_{s}=\left\{ \begin{array}{l} \mathrm{sign}(c_{i}^{\prime })(\left|c_{i}^{\prime }\right|-T),\;\left|c_{i}^{\prime }\right|>T\\ 0,\;\left|c_{i}^{\prime }\right|\leq T \end{array}\right.$$

where |·|, sign(·), and T indicate the absolute value, sign function, and threshold value, respectively. A fixed threshold $T=\sigma \cdot \sqrt{2\log(N)}$ (σ denotes data variance) was used in this study.

(6) For the remaining IMFs, denoised components ${\hat{c}}_{i}(t)$ can be obtained by repeating steps (3–5), and the final denoised signal $\hat{x}(t)$ is obtained by stacking the denoised components ${\hat{c}}_{i}(t)$, as follows:

(13) $$\hat{x}(t)={\hat{c}}_{i}(t)+\cdots +{\hat{c}}_{n}(t)+{\hat{c}}_{n+1}(t)$$

## 3. Synthetic Tests of EEMD-MSPCA-Based Denoising

**Figure 2:**
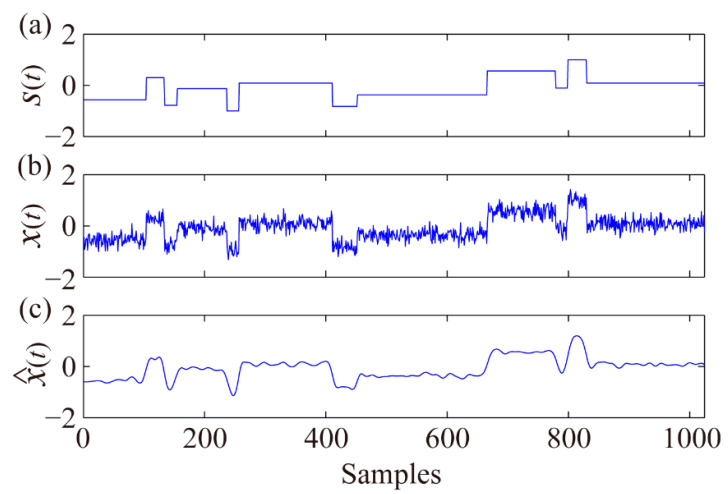
Real (**a**), noisy (**b**), and EEMD-MSPCA-denoised (**c**) Blocks signals.

Blocks, ECG, Bumps, Doppler shift, and Heavy sine signals with white Gaussian or random noise are usually adopted to test a denoising method, because it is easy to calculate the quantitative parameter signal to noise ratio (SNR) compared with a real signal. Thus, it is convenient for other researchers to reproduce or compare their results with ours. The Blocks signal [*s*(*t*) in Figure 2a] is selected to demonstrate the proposed denoising algorithm. The number of signal sampling points was set as *N* = 1024, and the added noise was expressed as *n*(*t*) = 0.2 × randn(1, 1024), i.e., the noise is 0.2 times the standard normal distribution, and the noisy signal *x*(*t*) is shown in Figure 2b.

**Figure 3:**
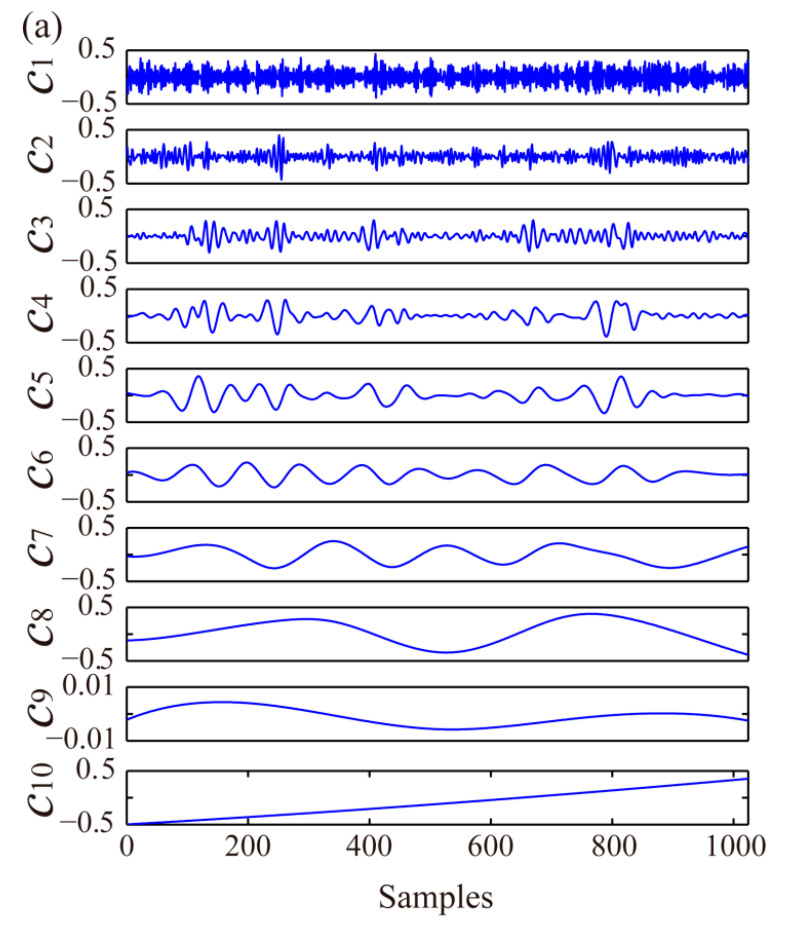

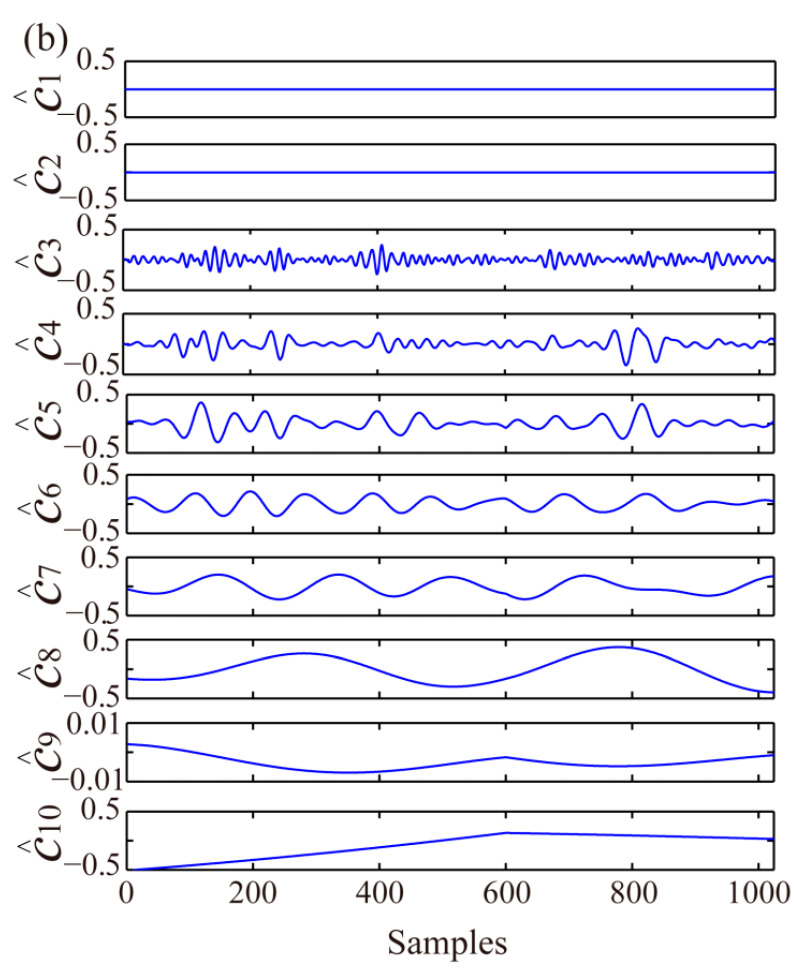
Original (**a**) and EEMD-MSPCA-denoised (**b**) IMFs for the noisy Blocks signal (Figure 2b).

With EEMD, *x*(*t*) is decomposed into nine IMF components, *c*_1_–*c*_9_, and one residual component, *c*_10_ (Figure 3a). These data indicate that the first few IMF components are mainly high-frequency noises, and the results obtained via Formula (11) show that the VCRs of the first two IMFs are <0.01. Therefore, they are eliminated (marked as zero vectors in Figure 3b) before PCA denoising.

**Figure 4:**
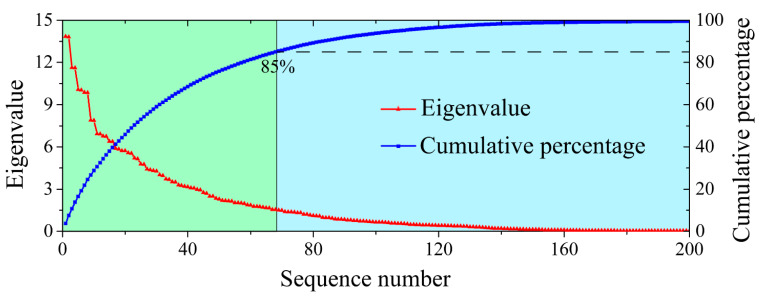
Descending ordered eigenvalues and cumulative eigenvalue percentages of the covariance matrix $H_{i}{}^{T}H_{i}$. The red points represent the series of eigenvalues arranged in descending order; the blue points indicate the series of cumulative eigenvalue percentages.

*c*_3_ was selected to illustrate the PCA-based denoising process. First, the Hankel matrix $H_{3}$ constructed with *c*_3_ using Formula (6) is decomposed into $H_{3}=U\sum V^{T}$ through SVD, with T=U∑ and P=V. Eigenvalues and eigenvectors of the covariance matrix $H_{3}{}^{T}H_{3}$ are calculated, and the eigenvalues are ranked in descending order (red points in Figure 4). The percentages of cumulative eigenvalues are shown in blue points in Figure 4. The score vectors corresponding to the cumulative eigenvalue percentages <85% are used for PCA-based *c*_3_ denoising, and the denoised signal ${c^{\prime }}_{3}$ is obtained by the reconstructed Hankel matrix ${\hat{H}}_{3}$, using Formula (10). Finally, the soft thresholding-denoised ${\hat{c}}_{3}$, derived from Formula (12), is shown in Figure 3b. By utilising the same process, the remaining components are denoised to obtain ${\hat{c}}_{4}\sim {\hat{c}}_{10}$.

As illustrated in Figure 3b, the denoised ${\hat{c}}_{3}$ enhanced the local amplitude characteristics of *N* = 100–250 and *N* = 300–450 and suppressed the noise amplitudes of other bands to an extent. Similar denoising results were obtained for the other IMFs, demonstrating the effectiveness of the EEMD-MSPCA-based denoising method. The final denoised signal (Figure 2c) was obtained using $\hat{x}(t)=\sum_{i=3}^{9}{\hat{c}}_{i}(t)+{\hat{c}}_{10}(t)$. SNR was selected to quantitatively evaluate the denoising performance, as defined in Formula (14). The SNRs increased from 6.99 to 12.55, and the denoised signal effectively retained the signal characteristics.

(14) $$SNR=10\mathrm{lg}[\sum_{i=1}^{N}s_{i}^{2}/\sum_{i=1}^{N}n_{i}^{2}]$$

where $s_{i}$ is the amplitude of the signal without noise and $n_{i}$ is the amplitude of noise, which is equal to the difference between $s_{i}$ and the denoised signal.

**Figure 5:**
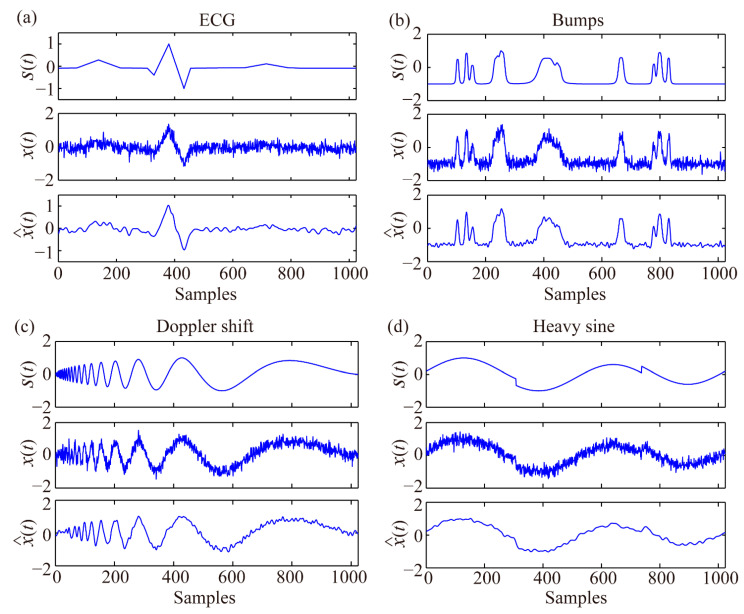
Four typical signals without noise (**top**), after adding noise (**middle**), and after EEMD-MSPCA denoising (**bottom**).

Furthermore, the ECG, Bumps, Doppler shift, and Heavy sine signals (separately shown top in Figure 5a–d) were selected to test the proposed denoising method. The noise is added using the same steps as those used in the Blocks signal test. The obtained noisy signals and EEMD-MSPCA-denoised signals are shown in the middle and bottom of Figure 5a–d, respectively, and their SNRs are listed in Table 1. Figure 5 depicts the local characteristics of the denoised signals, which are obviously enhanced, and Table 1 displays the increased SNRs of the denoised signals. A comparison of the proposed method with other denoising methods will be shown in the Discussion section.

## 4. MS Signal EEMD-MSPCA Denoising and P-Phase Arrival Picking

To further verify the effectiveness of the EEMD-MSPCA-based denoising method, 500 MS signals with a sampling frequency of 6000 Hz were randomly selected from the MS monitoring system in the Yongshaba mine (China) as the application dataset, which usually contains different noises (e.g., rock drilling, loco transportation, electrical noise, mine drawing noise, and blower vibration noise) and acts as a good dataset to test the denoising method. Because the real MS signal is unknown, we used the automatic P-phase arrival picking error instead of the signal SNR to evaluate the denoising performance. The STA/LTA, PAI-K, and AIC picking methods described previously [25] were employed, and the picking error was defined as the difference between the automatic and manual picking points; a smaller picking error represents better denoising performance.

**Figure 6:**
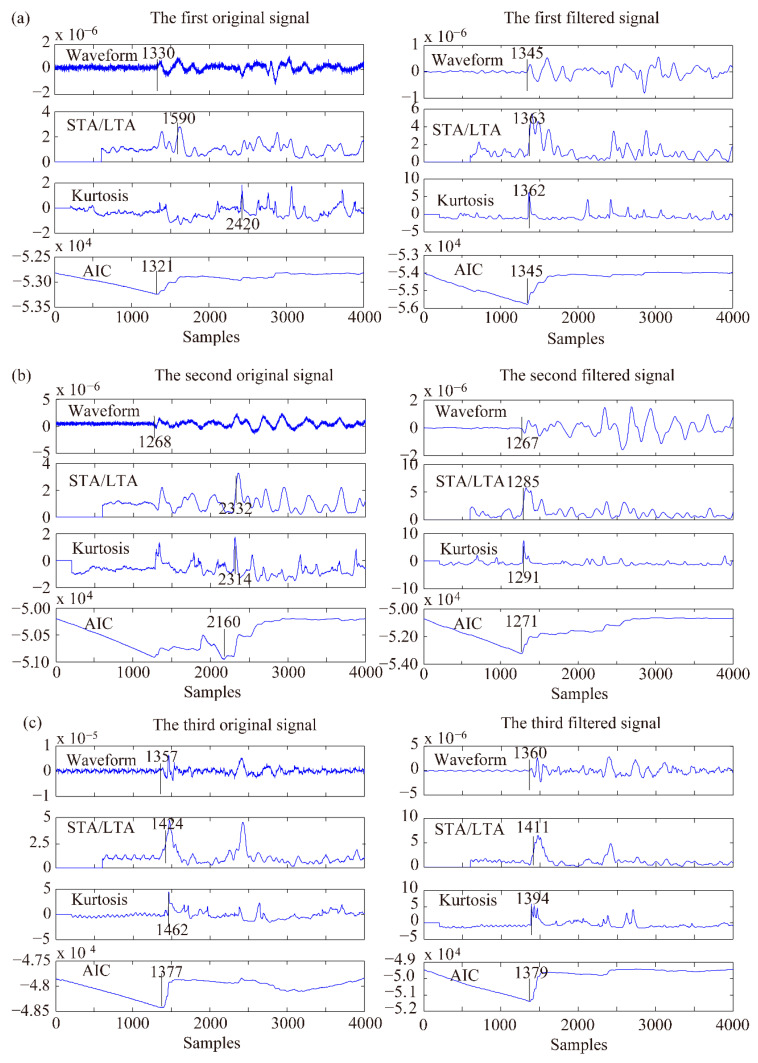
Three typical MS signals before and after EEMD-MSPCA denoising and their STA/LTA, PAI-K, and AIC pickings.

Three typical MS signals before and after denoising and the P-phase arrival picking results are shown in Figure 6. The original waveforms (Figure 6a–c) demonstrate that there is heavy noise in the recorded signals with low SNRs, especially for the third signal (Figure 6c), which includes high-frequency noise, power frequency noise, and spike noise. The EEMD-MSPCA-based denoising method can effectively remove the above-mentioned noises and retain the characteristics of P-phase arrival, which is favourable for P-phase arrival picking. The STA/LTA, kurtosis, and AIC values of the denoised signals are sharper than those without denoising, verifying the improvement of signal SNRs and the effectiveness of the proposed denoising method.

**Figure 7:**
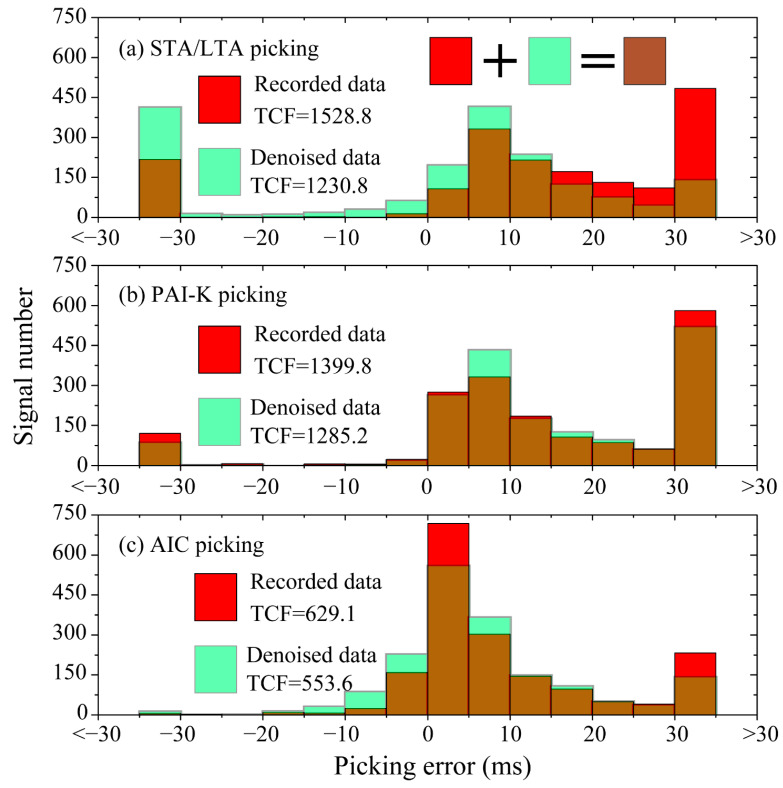
Picking error histogram and TCFs of different methods.

The picking error statistics of the STA/LTA picking, PAI-K picking and AIC picking for the 500 MS signals are shown in Figure 7a–c, respectively. The data indicate that there are more small picking error signals after the EEMD-MSPCA denoising, and the signal number of picking errors that are larger than 30 ms or no pickings is reduced, suggesting an improvement in SNR by the proposed denoising method. Furthermore, the total cost function (TCF) proposed by Li et al. [26] was selected for quantitative comparison. The cost function (*CF_i_*) and TCF are defined in Formulas (15) and (16), respectively, where the TCF is obtained through the summation of cost functions corresponding to P-phase arrival picking errors, and a small cost is incurred for a small picking error. Therefore, a smaller TCF indicates better picking results. Figure 7 shows that the TCFs of the denoised signals are smaller than those based on the recorded signals, and the TCF based on denoised signals and the AIC method showed the best picking results.

(15) $$CF_{i}=\{\begin{array}{cc} 0.0, & \mathrm{accuracy}<5\;\mathrm{ms}\\ 0.2, & 5\;\mathrm{ms}\leq \mathrm{accuracy}<10\;\mathrm{ms}\\ 0.4, & 10\;\mathrm{ms}\leq \mathrm{accuracy}<15\;\mathrm{ms}\\ 0.6, & 15\;\mathrm{ms}\leq \mathrm{accuracy}<20\;\mathrm{ms}\\ 0.8, & 20\;\mathrm{ms}\leq \mathrm{accuracy}<25\;\mathrm{ms}\\ 1.0, & 25\;\mathrm{ms}\leq \mathrm{accuracy}<30\;\mathrm{ms}\\ 1.5, & \mathrm{accuracy}\geq 30\;\mathrm{ms} \end{array}$$

(16) $$TCF=\sum_{i}CF_{i}$$

## 5. Discussion

**Figure 8:**
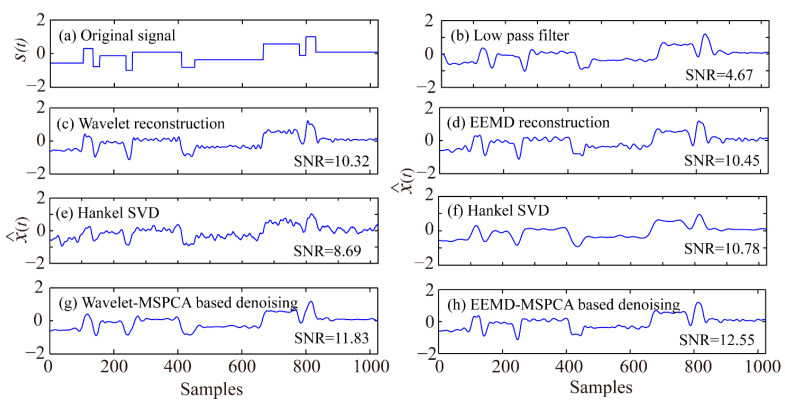
Denoising results of the noisy Blocks signal (Figure 2b).

To better validate the proposed denoising method, low-pass filter, wavelet reconstruction, EEMD reconstruction, Hankel–SVD, EEMD-Hankel–SVD, and wavelet-MSPCA-based denoising methods were selected for comparison. The filter used is a four-order low-pass Butterworth filter with cutoff frequency of 200 Hz, wavelet reconstruction and EEMD reconstruction both remove the first few detail coefficients with VCRs smaller than 0.01, Hankel–SVD was proposed by Jiang et al. [23], EEMD-Hankel–SVD was proposed by Li et al. [27], and the wavelet-MSPCA-based denoising was proposed by Bakshi [13]. Synthetic tests were performed on a Thinkpad laptop with 8 GB RAM and a 1.6 GHz Intel Core i5 Dual Core Processor. The results showed that the computational costs of the low-pass filter, wavelet reconstruction, EEMD reconstruction, Hankel–SVD, EEMD-Hankel–SVD, wavelet-MSPCA, and EEMD-MSPCA-based denoising methods were 0.01, 0.02, 0.25, 0.18, 0.15, 2.96, and 3.03 s, respectively. The EEMD has a relatively large computational cost because of the addition of white Gaussian noise in each EMD step. The EEMD-Hankel–SVD and proposed EEMD-MSPCA-based denoising methods require a much longer computation time than wavelet-MSPCA-based denoising, because the EEMD/wavelet decomposition times are proportional to the row number of the Hankel matrix shown in Formula (6). The denoising performance of the seven methods for the noisy Blocks signal (Figure 2b) is shown in Figure 8, and the SNRs of different denoising methods for the five noisy signals used in the synthetic tests are listed in Table 1.

Figure 8 demonstrates that the low-pass filter, wavelet reconstruction, EEMD reconstruction, Hankel–SVD, and wavelet-MSPCA-based denoising methods are inferior to the EEMD-MSPCA-based denoising method in some respects. Moreover, Table 1 shows that The SNR of the ECG signal improved from 0.49 to >8. Figure 5a shows that many amplitudes in the time series of the ECG signal are zero, causing the $\sum_{i=1}^{N}s_{i}^{2}$ and $\sum_{i=1}^{N}n_{i}^{2}$ in the SNR definition to be relatively small and large, respectively, after including noises, and a small SNR can then be obtained. Furthermore, there is a coefficient of 10 for the SNR definition. Thus, denoising can significantly improve the SNR in this case. Thus, the low-pass filter improves the waveform quality; however, it can decrease the SNR due to waveform shift, which is harmful for P-wave arrival picking. All the other denoising methods improved the SNRs, with the EEMD-Hankel–SVD and EEMD-MSPCA obtaining the best denoising performance. The underlying reasons are as follows: Hankel–SVD denoising removes information corresponding to low eigenvalues, while the other denoising methods eliminate high-frequency components in the denoising process, which illustrates the importance of removing high-frequency noise. Subsequently, EEMD reconstruction can reduce the mode mixing effect with a slightly superior denoising result compared to the wavelet reconstruction method. Finally, PCA- and soft thresholding-based denoising can denoise the remaining low-frequency noise [28], as well as retain the signal characteristics.

## 6. Conclusions

In this study, an EEMD-MSPCA-based signal denoising method was proposed to overcome the difficulty of wavelet-MSPCA in denoising a single signal. This method was verified using synthetic tests and MS signal denoising. The main conclusions are summarised as follows: (1) The proposed method eliminates high-frequency noise through EEMD and the variance contribution rate; subsequently, a Hankel matrix is constructed with each IMF for PCA denoising. PCA and soft thresholding can denoise low-frequency components, as well as retain signal characteristics. (2) EEMD-MSPCA-based denoising performs better than the low-pass filter, wavelet reconstruction, EEMD reconstruction, Hankel–SVD, EEMD-Hankel–SVD, and wavelet-MSPCA-based denoising methods. (3) EEMD-MSPCA-denoised signals effectively improve the P-phase arrival picking accuracy of the STA/LTA, PAI-K, and AIC methods, and the EEMD-MSPCA and AIC picking method achieves a good P-phase arrival picking result, which plays an important role in the MS parameter calculation (e.g., source location and focal mechanism). Further denoising improvements can consider other adaptive decomposition methods (e.g., VMD, local mean decomposition (LMD)), noisy component selection parameters (e.g., signal entropy and coefficient), and combining with other denoising methods.

## Figures and Tables

**Table 1:** SNRs of the five noisy signals with different denoising methods.

Signal Name	Noisy Signal	Low-Pass Filter	Wavelet Reconstruction	EEMD Reconstruction	Hankel–SVD	EEMD-Hankel–SVD	Wavelet-MSPCA	EEMD-MSPCA
Blocks	6.99	4.67	10.32	10.45	8.69	10.78	11.83	12.55
ECG	0.49	4.46	8.32	8.63	2.93	9.68	9.26	9.43
Bumps	12.55	6.29	17.83	18.38	14.93	19.23	19.88	20.13
Doppler shift	9.32	6.16	15.18	15.59	11.46	16.51	16.58	16.84
Heavy sine	9.51	15.95	18.52	18.67	14.87	19.18	19.04	19.28

## Data Availability

The data is available through 222.85.135.26:8001/ims-synapse.
